# Neural networks for link prediction in realistic biomedical graphs: a multi-dimensional evaluation of graph embedding-based approaches

**DOI:** 10.1186/s12859-018-2163-9

**Published:** 2018-05-21

**Authors:** Gamal Crichton, Yufan Guo, Sampo Pyysalo, Anna Korhonen

**Affiliations:** Language Technology Laboratory, TAL, University of Cambridge, 9 West Road, Cambridge, CB39DB UK

**Keywords:** Link prediction, Neural networks, Data mining, Literature-based discovery, Drug-target interaction

## Abstract

**Background:**

Link prediction in biomedical graphs has several important applications including predicting Drug-Target Interactions (DTI), Protein-Protein Interaction (PPI) prediction and Literature-Based Discovery (LBD). It can be done using a classifier to output the probability of link formation between nodes. Recently several works have used neural networks to create node representations which allow rich inputs to neural classifiers. Preliminary works were done on this and report promising results. However they did not use realistic settings like time-slicing, evaluate performances with comprehensive metrics or explain when or why neural network methods outperform. We investigated how inputs from four node representation algorithms affect performance of a neural link predictor on random- and time-sliced biomedical graphs of real-world sizes (∼ 6 million edges) containing information relevant to DTI, PPI and LBD. We compared the performance of the neural link predictor to those of established baselines and report performance across five metrics.

**Results:**

In random- and time-sliced experiments when the neural network methods were able to learn good node representations and there was a negligible amount of disconnected nodes, those approaches outperformed the baselines. In the smallest graph (∼ 15,000 edges) and in larger graphs with approximately 14% disconnected nodes, baselines such as Common Neighbours proved a justifiable choice for link prediction. At low recall levels (∼ 0.3) the approaches were mostly equal, but at higher recall levels across all nodes and average performance at individual nodes, neural network approaches were superior. Analysis showed that neural network methods performed well on links between nodes with no previous common neighbours; potentially the most interesting links. Additionally, while neural network methods benefit from large amounts of data, they require considerable amounts of computational resources to utilise them.

**Conclusions:**

Our results indicate that when there is enough data for the neural network methods to use and there are a negligible amount of disconnected nodes, those approaches outperform the baselines. At low recall levels the approaches are mostly equal but at higher recall levels and average performance at individual nodes, neural network approaches are superior. Performance at nodes without common neighbours which indicate more unexpected and perhaps more useful links account for this.

**Electronic supplementary material:**

The online version of this article (10.1186/s12859-018-2163-9) contains supplementary material, which is available to authorized users.

## Background

The biomedical domain has a wealth of datasets which encapsulate varied, useful information and can be represented as graphs. It is useful to know if any information is missing from these or what information may be added to them in the future. Link prediction is the task of proposing links which are not currently part of a graph but should be or could become a part of it. If the information in these datasets are represented as graphs, link prediction has application in various biomedical information processing tasks. These include predicting Drug-Target Interactions (DTI) for drug re-purposing, predicting Protein-Protein Interactions (PPI), facilitating Literature Based Discovery (LBD) for generating hypotheses from publications and automating knowledgebase completion.

Link prediction has been used for predicting DTI by applying it to graphs representing drugs/chemicals and the proteins which they interact with [1, 2]. It has also been used to facilitate LBD by applying it to bibliographic networks [3, 4] and term co-occurrence networks [5]. Kastrin et al. [6] also used it on MeSH [7] to demonstrate its use on graphs of organised knowledge. Grover and Leskovec [8] used it to predict PPI from a subset of the BioGRID graph [9].

Some of these methods do not make use of the information contained in the structure of graph, which can aid in link prediction. Others which do use this information either do so using approaches which are only able to draw a limited amount of patterns from the graph or provide restricted datasets to their methods. This work makes use of information in the graph by using methods which are able to extract non-linear patterns from graph structure and use this information to predict the likelihood of a link forming between two nodes.

This is possible in large part to the recent rise in the number of works using various neural networks to embed graphs in low-dimensional spaces. These produced vectors of real numbers which are representations of a graph’s nodes that aim to place similar nodes close to each other in the vector space and dissimilar ones far apart based on the structure/topology of the graph. These vectors are called *embeddings* and the methods that create them include DeepWalk [10], node2vec [8], LINE [11], SDNE [12] and HOPE [13].

These opened the possibility of using rich representation as inputs to neural link predictors which output how likely it is for a link between two nodes to form. Several works have already begun to explore this avenue and report promising results, however their approaches have not comprehensively addressed the issues of using these methods for link prediction. Particularly lacking are experiments in realistic settings like time-slicing, where graphs are split so that predictors are evaluated on how well they predict chronologically later links, and evaluating performances with metrics where all nodes have equal weight as link prediction applications may need to perform well across most nodes as opposed to fewer hub nodes.

In this work we employed four graph embedding algorithms: DeepWalk, LINE, node2vec and SDNE. We investigated how a neural predictor, using representations from these methods, performs on link prediction in biomedical graphs containing information which can be used for several bioinformatics tasks including DTI, PPI and LBD. We compared this approach to the performance of established baseline methods Common Neighbours (as used in [14]), Adamic-Adar [15] and Jaccard Index [16]. These methods were chosen because they continue to be very competitive and challenging baselines for link prediction [12, 17], are conceptually simple and scale well to large graphs.

We report results on graphs which represent real biomedical information in settings where links were randomly removed as well as where links were removed by time-slicing. These results are evaluations with metrics that weigh the performance at each node equally and those which do not as they illustrate different aspects of a predictor’s performance and can be useful depending on its application. These contributions together provide large-scale comparisons and analyses that inform and explain the best approaches to link prediction and highlight areas of further research.

The “Related work” section details related works and gives necessary background information. The “Important considerations” section presents some factors which affect link prediction experiments and thus interpretability and applicability of results. Details of the models, methods and datasets used are in the “Methods” section. Our experimental setup is given in the “Experiments” section. We analyse the results and their implications in the “Results and discussion” section. The “Conclusions” section concludes the work and gives possible future directions.

### Related work

#### Link prediction in general and biomedical domains

Liben-Nowell and Kleinberg [17] first formulated the link prediction problem in social networks. Existing link prediction works have mostly focused on determining which links will form next in various social networks. These links can represent friendships [18, 19], collaborations and co-authorships [19, 20], citations [21] and online transactions [21] among others. Link prediction has also been used on large-scale knowledge-bases to add missing data and discover new facts [22, 23].

Katukuri et al. [3] used supervised link prediction on a large-scale biomedical network of concept co-occurrence in documents to generate hypotheses. They used manually-created features to predict links which represented hypotheses in a time-sliced corpus. Wang and Zeng [1] performed link prediction for proposing DTI using Restricted Boltzmann Machines (RBMs). Lu et al. [2] used similarity indices, such as Common Neighbours and Katz Index, to predict links in a DTI network.

#### Node representations as embeddings

Graphs encode knowledge and can be processed to extract information which may not be easily seen before. For a machine to perform this processing, the graph must be represented in a format which it can use, usually by representing nodes as vectors of real numbers. Works on node representation aim to devise methods which can create vector representations which preserve the original information in the graph. In general the information in a graph can be classified as first or second (or higher) order proximity [11, 24].

**Fig. 1:**
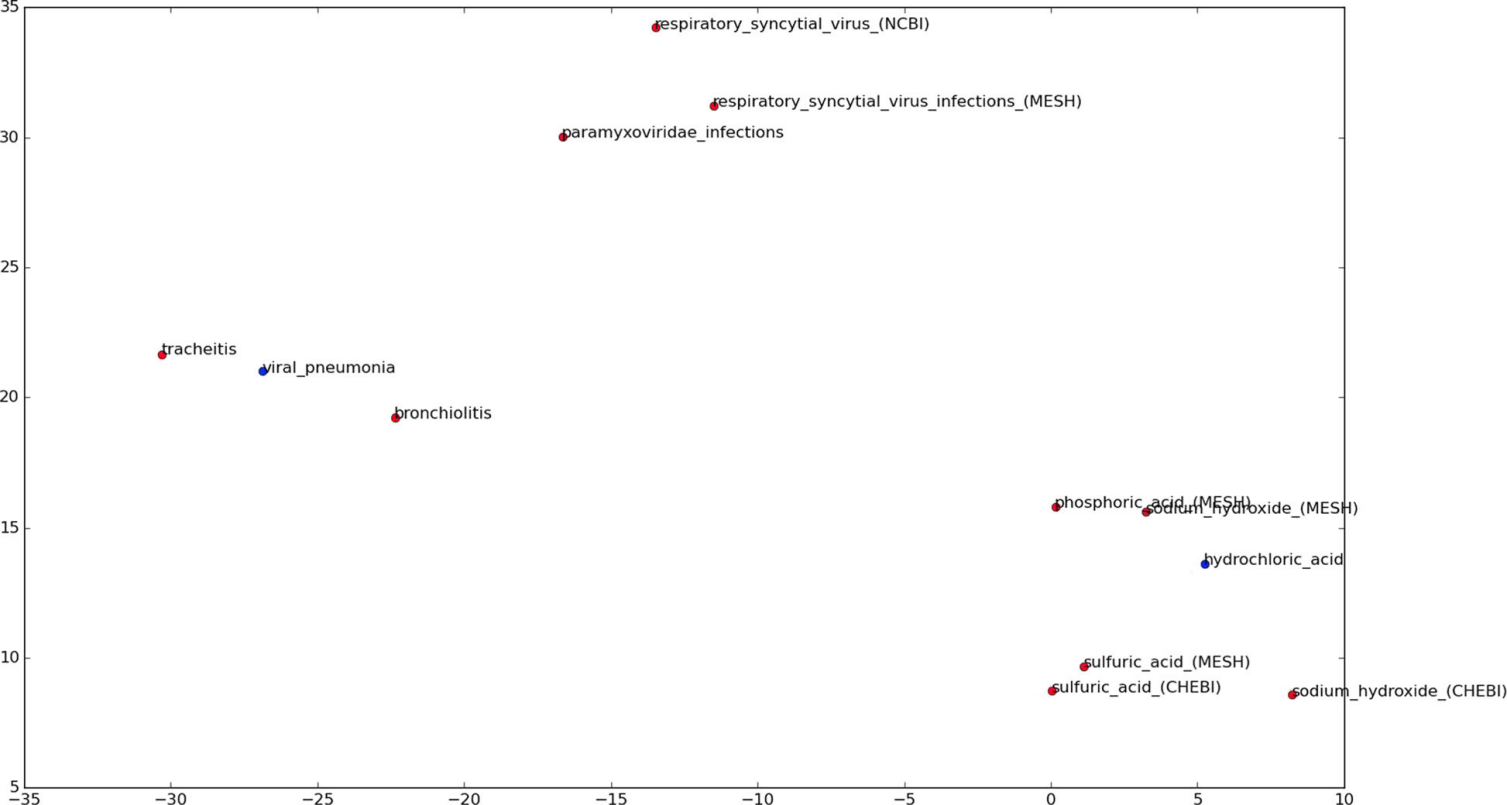
Visualisation of ‘Viral Pneumonia’ and ‘Hydrochloric Acid’ from PubTator dataset. Nodes representing respiratory infections are close to the former while those of acids and other chemicals are close to the latter

Given two nodes in a graph, first order proximity is concerned with the strength of the direct link between them. Second order proximity between two nodes compares their neighbourhoods and classes them as similar if their neighbourhoods are similar. The extent to which a method can preserve the proximities of a graph when creating representations determines its quality. The node representations created by recent research models each node as a vector in a space where similar nodes are located close to each other. These vectors are often called node *embeddings* and referred to in this work as such. Figure 1 visualises a portion of this vector space for one of the datasets used in this work created with one of the methods used. There has been a proliferation of methods which seek to create these node embeddings from graphs and it would be unwieldy to include all of them in this work, so we utilise four of the most popular ones whose implementations are freely available online.

##### DeepWalk

[10] uses random walks on graphs to learn latent representations of nodes and encodes them in a continuous space. It does this by treating random walks on graphs like sentences in a natural language and generalizes recent advancements in language modeling [25] developed for word sequences to graphs. This makes it easy to use existing language modeling tools to implement, but it consequently lacks an objective function which explicitly captures the graph’s structure.

##### Large-scale information network embedding (LINE)

[11] explicitly defines two optimization functions to capture the structure of the graph. One captures first order proximity and the other captures second order proximity. They report that training their model with each setting then concatenating the outputs gave the best performance.

##### Node2vec

[8] is similar to DeepWalk in how it preserves higher order proximity between nodes. It does so by maximizing the probability of the occurrence of subsequent nodes in random walks over a graph. The difference to DeepWalk is that node2vec’s random walks are parameterized to provide a trade-off between prioritising breadth-first or depth-first walks. Choosing the right balance enables node2vec to preserve first- and second-order proximity between nodes to potentially produce more informative walks, leading to superior embeddings.

##### Structural deep network embedding (SDNE):

[12] argue that the shallow models which the other methods use cannot adequately capture the highly non-linear structure of most graphs. Since deeper models have proven successful at capturing non-linearity in complex data, they use them to create representations. Their model jointly optimises unsupervised and supervised parts. The unsupervised part produces an embedding for a node which can reconstruct its neighborhood. The supervised part applies a penalty when nodes deemed to be similar are mapped far from each other in the vector space.

#### Node embeddings for link prediction

There have been several works which used the embeddings created from neural network methods for link prediction. The evaluation metrics mentioned here are explained in the “Meaningful evaluation metrics” section. To the best of our knowledge, none of these works included time-sliced datasets.

Grover and Leskovec [8] evaluated node2vec embeddings on three graphs, including a PPI subset of BioGRID, and compared the results to Common Neighbours, Jaccard Index, Adamic-Adar and Preferential Attachment. This work evaluated using Area Under the Receiver Operator Characteristics Curve and its largest graph contained 19,706 nodes and 390,633 links.

Wang et al. [12] used the embeddings created from SDNE on a single dataset of 5242 nodes and 28,980 links. They compared to LINE, DeepWalk, GraRep, Laplacian Eigenmaps and Common Neighbours. They evaluated using precision at *k* for the full network and Mean Average Precision (MAP) for a sparse version of the graph.

Ou et al. [13] performed link prediction on two graphs to compare performance of HOPE to Partial Proximity Embedding, LINE, DeepWalk, Common Neighbours and Adamic-Adar. The larger graph had 834,797 nodes and 50,655,143 links. They randomly sampled 0.1% of node pairs for evaluation but the amount used for creating embeddings is not reported. They evaluated using precision at *k.*

Goyal and Ferrara [24] compared the performances of Laplacian Eigenmaps, Graph Factorization, node2vec, SDNE and HOPE to perform link prediction on four datasets including a PPI subset of BioGRID. They evaluated using precision at *k* and MAP to determine how performance corresponded to changes in vector dimensions. They experimented on five random subsets of each graph created such that each subset contained 1,024 nodes.

### Important considerations

This section presents some factors which affect link prediction experiments and thus the interpretability and applicability of their results. To the best of our knowledge, no previous study using node embeddings for link prediction has taken all of these factors into consideration.

#### Link prediction setting

There are two main link prediction settings. In random-slicing, a percentage of the links are removed randomly and evaluation consists of predicting the removed links. Time-slicing (or literature-slicing) aims to take the temporal evolution of the graph into account and only links formed after some point in time, *t*, are removed. The state of the graph before *t* is given to the link predictor and its aim is to predict links formed at a later time. The first setting is applicable when the current knowledge represented by the graph is incomplete and link prediction aims to complete it as well as when the temporal data for the graph is unknown or irrelevant. The second can be used to predict the future state of the graph and so can suggest feasible links to investigate. This setting can make link prediction more challenging for two reasons: 1) new nodes can be introduced to the graph at later time periods which will present little or no information to the link predictor to use as these nodes will have no links to other nodes in the time period which the predictor uses to make predictions and 2) in evolving graphs, the easier links tend to form before more difficult ones, so the links to be predicted in later time periods tend to be more difficult.

#### Meaningful evaluation metrics

Several metrics which measure different aspects of the predictor’s performance have been used to evaluate link prediction methods. It is useful to distinguish between metrics which weigh all nodes in the network equally and metrics which do not. We refer to the former as an node-equality metrics and the latter as link-equality metrics. Node-equality metrics can be robust to performance at hub nodes, which tend to be easier for link prediction, and some link prediction applications are more concerned with how a predictor performs across a cross-section of nodes than how many links it predicts across the entire graph. This is analogous to the difference between micro- and macro-averaging. The following metrics were used in this and previous works. In-depth explanations of these metrics can be found in several works including [24, 26].

##### Area under the precision-recall curve:

Recall measures what percentage of positives were returned. Precision measures what percentage of the results are true positives. These metrics are used to construct a Precision-Recall Curve which illustrates how the increase in recall affects precision. The area under this curve is a link-equality metric.

##### Area under the receiver operating characteristics curve:

True positive rate is equivalent to recall. The false positive rate measures how many negatives were returned as false positives by the predictor. These metrics are used to construct a Receiver Operating Characteristics (ROC) Curve which illustrates this relationship. The area under this curve is a link-equality metric.

##### Precision at *k*:

The above metrics measure performance across all recall levels but some uses of link prediction are only interested in the quality of highly ranked results. Precision at *k* or the top *k* predictive rate is the percentage of true positives among only the top *k* ranked links. This is a link-equality metric.

##### Mean average precision (MAP):

Given a ranked list of predicted links relevant to a particular node, we calculate the precision after each true positive. The average of these values gives the average precision for that node. This done over all nodes in the graph gives a single value, node-equality measure.

##### Averaged R(elevant)-precision:

Similar to MAP but instead of calculating the precision after each positive link in the list of results for a given node, precision is only calculated with the top *R* results. *R* is determined by how many true positives exist for the node. The main difference from MAP is that this metric does not consider the remainder of the ranked list outside of the top *R*. This also gives a single value, node-equality measure.

#### Scalability, sparsity and negatives

Biomedical and other real-world graphs reflect complex relationships between numerous entities so methods employed to make use of them must be able to scale, usually to hundreds of thousands of nodes and millions, or billions of links.

Supervised machine learning approaches require both positive and negative examples to train models. Graphs tend to be sparse as only a fraction of potential links are actually formed. While a link between two nodes in a graph confirms a relationship, the absence of a link does not confirm a lack of relationship. The assumption that most node pairs which do not have a link have no relationship is not always true. This means that these links can potentially be used as negative examples in supervised machine learning techniques for link prediction. In real-world situations, the model will inevitably encounter such links and it will be trained on some negative examples which would later turn out to be positive.

Due to the problems of large size and extreme sparsity, it is usual to create negatives for training and testing by sub-sampling from the list of potential negative links. The manner in which this sub-sampling is done can affect the performance of the link predictor. Yang et al. [26] looked in great detail into these issues and how they can affect link prediction evaluation. The issue of scalability also affects the ratio of negative to positive examples in the evaluation data. In real-world situations unformed links far outweigh the formed ones, but it is often computationally prohibitive to replicate the real positive to negative ratio.

#### Node combination method

A neural network approach to link prediction with node embeddings requires the model input to be a single vector so the embeddings of the nodes involved in a link need to be combined. This can be done in several ways which can affect the predictor’s performance. Concatenating the embeddings is simple and preserves all information but doubles the size of the input. Grover and Leskovec [8] used four methods which preserve the input size and we experimented with all five methods, detailed in Table 1.

**Table 1:** Node Combination methods. Binary operators are element-wise

Operator	Definition
Average	$\frac {f_{i}(u) + f_{i}(v)}{2}$
Concatenate	*f*(*u*). *f*(*v*)
Hadamard	*f*_*i*_(*u*)∗*f*_*i*_(*v*)
Weighted-L1	|*f*_*i*_(*u*)−*f*_*i*_(*v*)|
Weighted-L2	|*f*_*i*_(*u*)−*f*_*i*_(*v*)|^2^

## Methods

### Datasets

The graphs we use were created from the following datasets. The graph details can be found in Table 2.

**Table 2:** The datasets and their relevant details (undirected link count)

	Node	Link	Has	Link
Dataset	count	count	dates	type
BioGRID	65,026	1,076,308	Yes	Published
				interactions
MATADOR	3,704	15,843	No	Drug-target
				interactions
PubTator	265,148	6,854,054	Yes	Literature co-
				occurrences

**Manually Annotated Target and Drug Online Resource (MATADOR):** This is an open online DTI database [27]. It includes interaction between chemicals and proteins. Following [2] the Chemical and Protein IDs are used to form a bipartite DTI graph. Thus the links in this graph represent interactions between chemicals and proteins representing drugs and targets respectively.

**Biological General Repository for Interaction Datasets (BioGRID):** This is an open database created from manually curating experimentally-validated genetic and protein interactions that are reported in peer-reviewed publications [9]. The latest release [28] includes over 1 million Genetic and Protein interactions across all major organism species and humans. Links in this graph represent biomedical interactions from published, experimentally-validated genetic and protein interactions, including PPI. We use version 3.4.147 of this dataset.

**PubTator:** Biomedical entities recognised by PubTator [29] mentioned in the titles and abstracts of PubMed publications from 1873 to 2017 were used to create this dataset. A link exists between two biomedical entities if they co-occur in a single sentence. The annotations were downloaded on June 20th, 2017.

### Settings for training node representation methods

The hyper-parameter settings for DeepWalk and LINE were the same as used in [12] which is a recent work which compared both of those methods. Parameters for node2vec which overlapped with DeepWalk’s were set to the same values. All methods created embeddings of 100 dimensions as this was determined to be a good value on datasets which are not used as part of this work.

**DeepWalk:** window size = 10, walk length = 40, walks per vertex = 10. **LINE:** learning rate = 0.025, number of negative samples = 5 and total number of samples = 10 billion. According to [11], LINE performs best when it is run twice to obtain first- and second-order proximity embeddings which are concatenated and L2 normalized. We follow their recommendations. For each order we created half the number of dimensions as needed so that we had the appropriate number when concatenated. **node2vec:** window size, walk length and walks per vertex were the same as DeepWalk’s. The parameters p and q were 2 and 4 respectively as randomly chosen from the optimal set given by the creators [8]. We used **SDNE** implementations from both [24] and [12] with hyperparameters as used by [24]: *α* = 1e-6, *β* = 5, *ρ*= 0.3, *xeta* = 1e-4 and *nu1 & nu2* = 1e-3.

### Neural link predictor and baselines

The neural link predictor was a binary classifier implemented as a feed-forward neural network with a single hidden layer containing 100 Rectified Linear Units [30]. It accepted the vector representation of two nodes representing a link by combining their individual vector representations with operators defined in Table 1 and output the probability of a link forming between the nodes. These probability scores were used to create a ranked list of all links in the evaluation set. The model was trained for 7 epochs. This minimalist model was chosen so that the contribution from each node embedding method could be compared without the confound of the contribution of a powerful neural network model. The other parameters were determined to be a good values based on datasets which are not used as part of this work.

We employed three baseline methods which have been used successfully for link prediction: Adamic-Adar, Common Neighbours and Jaccard Index. It is necessary to modify these slightly for bipartite graphs following [31]. Their definitions are in Table 3.

**Table 3:** Baseline methods for node pair *(u, v)* with neighbour sets *N(u)* and *N(v)*. $\hat {N}$*(x)* are the neighbours of the neighbours of *x*

		Bipartite
Name	Definition	definition
Adamic-Adar	$\frac {1}{log(|N(u) \cap N(v)|)}$	$\frac {1}{log(|N(u) \cap \hat {N}(v)|)}$
Common Neighbours	|*N*(*u*)∩*N*(*v*)|	$|N(u) \cap \hat {N}(v)|$
Jaccard Index	$\frac {|N(u) \cap N(v)|}{|N(u) \cup N(v)|}$	$\frac {|N(u) \cap \hat {N}(v)|}{|N(u) \cup \hat {N}(v)|}$

### Experiments

We experimented with both link prediction settings explained in the “Link prediction setting” section where possible. For the MATADOR dataset, there was no temporal data so no time-sliced experiments could be done.

The existing links of each graph were split into 3 segments whose details follow. For the random-slice experiments, 60% of the links were used to create the node embeddings, 10% was used to train the neural link predictor where necessary and the remaining 40% were used to evaluate the predictors. The data used to train the model was also used to create the embeddings since there is no reason to withhold that information from the node representation methods and more information will lead to better representations. The test set is larger than is usually found in machine-learning works but being able to demonstrate good results with reduced training data is a desirable quality. For time-slice experiments, we sought to have similar split sizes as the random-sliced, but exact sizes were not possible as it depends on the amount of links in a year. The details of the time slices are in Table 4. For both settings, after splitting the existing links, we then sub-sampled negative examples by randomly sampling from all the possible node pairs without a link while maintaining a 1:1 ratio of positive to negative links. Following [8], graph connectivity was maintained in the random-sliced data, but this was not possible to enforce in the time-sliced data as the links in each slice were determined by what year they were added to the dataset. Due to the varying sizes of the graphs, for precision at *k* we let the total amount of positives which can be returned dictate the *k*. We report *k* to be 30% of all possible positives here. Results on additional *k* values can be found in the Additional file 1. We implemented the baselines listed in the “Neural link predictor and baselines” section and used them on the same induction, train and evaluation subsets. We used Scikit-learn [32] to efficiently calculate most of the metrics on the predictions of the models.

**Table 4:** Time-sliced details (Note: Induction includes Train)

	Link	Time	Link	Link
Dataset	use	slice	count	percentage (%)
BioGRID	Induction	1970-2014	678,994	63.08
	Train	2013-2014	121,442	11.28
	Test	2015-2017	397,302	36.91
PubTator	Induction	1873-2003	4,069,683	59.38
	Train	2001-2003	614,031	5.90
	Test	2004-2017	2,784,371	40.62

## Results and discussion

The scores presented in the result tables are the means of three runs of each experimental setting. Scores in **bold** represent the best score for a particular metric. The best score and all other scores were tested for statistical significance using a two-tailed *t*-test with *α* = 0.05. Scores with an asterisk (*) are not significantly different from the best score, scores without an asterisk are significantly different. The standard deviation of the means reported here were excluded to aid readability but can be found in the full result tables in the Additional file 1 which accompanies this paper.

The performance of the neural classifier with inputs combined using Hadamard, Weighted-L1 and Weighted-L2 are not the best performers in any experiments so they are left out of the tables in this section. The results for embeddings created with SDNE are much poorer than the others and are left out of these tables for space considerations. The full set of results containing these figures can be found in the Additional file 1. It also contains analysis about interesting results involving DeepWalk embeddings combined with Weighted-L1 and -L2. The most efficient reference implementations of SDNE available exceeded our computational resources for the BioGRID and PubTator graphs, so we report no results for them in those settings.

### MATADOR

These results are in Table 5. The Common Neighbours and Jaccard Index baselines are the best performers across all metrics. This can be attributed to the graph being too small for the neural network methods to create good embeddings for each node which lead to poor input to the neural link predictor. For precision at *k*, averaged and concatenated DeepWalk embeddings also produce comparable results. Adamic-Adar performs the worse of the baselines despite the fact that it is common neighbours-based. This is because the algorithm weighs a small amount of shared items between entities high and a higher amount of shared items less. As we are only using amount of common neighbours as the shared item between two nodes here, links which score high for common neighbours will score lower for Adamic-Adar.

**Table 5:** MATADOR random-slice results

	Node	AUC	AUC		Avg.	Prec
Method	combination	(ROC)	(PR)	MAP	R-prec	@ *k*
Deep-	Average	95.93	95.82	89.81	86.86	98.77*
Walk	Concat	94.97	94.83	88.30	84.63	98.34*
LINE	Average	80.63	81.30	67.74	61.04	91.65
	Concat	81.16	81.82	68.53	61.42	92.00
node-	Average	78.38	78.75	66.42	59.32	88.67
2vec	Concat	77.62	77.54	65.44	58.40	87.25
AA	N/A	91.97	88.40	87.16	85.06	86.87
CN	N/A	**97.27**	97.04*	**95.47**	**94.64**	98.74*
JI	N/A	97.23*	**97.10**	94.72	92.29	**98.96**

(Bold: best score, *: not statistically different from best)

### BioGRID

**Random-slice:** The results of this experiment are in columns 3-7 of Table 6. Concatenated and averaged node2vec embeddings are the best performers across 4 of the 5 metrics and the best performer in the remaining metric is not significantly better. Averaged LINE embeddings are not significantly different from the best performer in any metric. In general the neural network approaches outperform the baselines. This is not surprising as it is a favourable condition for the neural network methods: there is a large amount of data to induce the node embeddings with and, since connectivity is guaranteed, all nodes have a chance of getting an embedding which is better than its random initialization. These embeddings would then perform better in the neural link predictor.

**Table 6:** BioGRID random-slice and time-slice results

		Random slice					Time slice				
	Node	AUC	AUC		Avg.	Prec	AUC	AUC		Avg.	Prec
Method	combination	(ROC)	(PR)	MAP	R-prec	@ *k*	(ROC)	(PR)	MAP	R-prec	@ *k*
Deep-	Average	97.69	97.62	79.24	73.86	99.30	89.40	90.10	68.94	63.30	97.25*
Walk	Concat	97.74	97.65	82.48	77.70	99.18	92.12	92.78	71.61	65.96	98.04
LINE	Average	98.10*	97.80*	83.13*	78.22*	99.54*	91.86	92.31	72.85	67.76	97.40
	Concat	98.08	97.76	82.94	78.04	99.29	93.55	93.74	73.60	68.57	97.90
node-	Average	98.32*	97.97*	85.70*	81.17*	99.38*	**95.25**	**95.43**	74.91	**70.39**	98.26
2vec	Concat	**98.51**	**98.26**	**86.49**	**81.84**	99.49*	93.66	94.66*	73.48	68.77	98.40*
AA	N/A	86.10	90.75	70.97	57.65	96.13	77.46	87.69	74.84	61.39	98.10
CN	N/A	91.20	94.96	75.72	69.81	**99.64**	85.07	91.81	**76.20**	67.73	**99.38**
JI	N/A	90.80	93.95	73.93	68.79	98.59	84.74	90.20	75.60	67.49	97.45

(Bold: best score, *: not statistically different from best)

Common Neighbours is the best performer for precision at k, although it is not significantly better than four neural network approaches. The chosen k focuses only on the very highly ranked links and other works such as [2] have already posited that Common Neighbours returns good results at the top of its ranked list. Its failure to perform well for the AUC metrics highlights that performance degrades substantially lower in its ranked list of links. Its poor performance at the node-level metrics also indicate that the links which it is predicting correctly at the top of its ranking are dominated by the links of hub nodes.

**Time-slice:** These results are in columns 8-12 of Table 6. Averaged node2vec embeddings are the best performer for three of the metrics and embeddings combined by concatenation are not significantly worse in two of the metrics. Common Neighbours performs the best in two metrics, including one node-level metric where it is significantly better than all other approaches. In general, the performances of Common Neighbours and Jaccard Index are not as far behind that of the neural network approaches as they are for the random-sliced setting of this dataset. This is due to a property of the dataset: it is skewed towards later publications. Because of this bias, when it is split by time-slicing, 14.5% of the nodes representing entities in the test slice had never occurred in the induction slice. This means that the neural network approaches could not create good embeddings for them so they are simply assigned their randomly initialized values which negatively influenced the predictor’s performance.

It is interesting that the best performer for each of the node-level metrics is different and the difference between them is significant in each case. This indicates that the neural predictor using averaged node2vec embeddings is good at ranking true positives for a given node within the top *R* while Common Neighbours is better at ranking more positives at the very top of the lists but does not capture some of these positives.

### PubTator

**Random-slice:** These results are in columns 3-7 of Table 7. Concatenated DeepWalk embeddings produce the best results in three of the metrics and is not significantly worse in another. Averaged and concatenated LINE embeddings are on par with the best results except in a single instance.

**Table 7:** PubTator random-slice and time-slice results

		Random slice					Time slice				
	Node	AUC	AUC		Avg.	Prec	AUC	AUC		Avg.	Prec
Method	combination	(ROC)	(PR)	MAP	R-prec	@ *k*	(ROC)	(PR)	MAP	R-prec	@ *k*
Deep-	Average	98.85	99.01	83.67	75.97	99.93*	93.86*	95.51*	70.78*	62.16*	**99.89**
Walk	Concat	**99.20**	**99.30**	**91.01**	85.46	99.94*	**93.99**	**95.70**	**71.11**	**62.65**	**99.89**
LINE	Average	99.10*	99.23*	90.36*	84.56	**99.97**	88.68*	92.27*	55.61*	46.41*	**99.89**
	Concat	99.13	99.24	90.07	84.03	99.95*	90.32	93.01	62.51	53.21	**99.89**
node-	Average	98.71	98.90	82.98	75.29	99.94*	88.40	92.07	55.72	46.48	99.87
2vec	Concat	99.16	99.21	88.94	82.14	99.92*	88.13	91.83	53.24	43.69	99.84
AA	N/A	92.92	84.56	56.48	66.38	83.33	85.10	80.24	35.49	40.13	90.56
CN	N/A	98.40	98.28	79.84	**87.10**	99.94*	88.37	88.83	43.67	46.59	99.84
JI	N/A	92.36	87.59	65.44	59.74	91.21	86.08	83.52	38.66	38.75	94.27

(Bold: best score, *: not statistically different from best)

An interesting result is that Common Neighbours performs the best for averaged R-precision in addition to its performance for MAP being significantly worse than the best. These indicate that it captures several true positives for a given node within the top *R* but not rank them at the top of that list and is prone to ranking some of the true positives quite low. The approaches which outperform it for MAP but not for averaged R-precision are better at ranking true positives just outside of the top *R* than it is.

**Time-slice:** These results are in columns 8-12 of Table 7. Similar to the random-sliced experiments on this dataset, concatenated DeepWalk vectors produce the best results in all metrics although there is a four-way tie for precision at k. Averaged LINE embeddings are on par with the best results here as well. The neural network approaches vastly outperform the baselines. This is noteworthy as this is the largest graph, in a difficult realistic setting and with no apparent biases to hinder the neural network methods.

### General

We hypothesize that the superior performance of the neural network methods are due to the limitations in recall of Common Neighbours and baselines based on it. It is possible for links to form between nodes which have no previous common neighbours and these methods would fail in such cases. We investigated this limitation and the effect it has on the performance of the link predictors. We first quantified these links in the test examples of each experimental setting then looked at how the best predictors in each category ranked these links. In the latter, we specifically looked at whether the links were ranked in the top or bottom half of the overall ranked lists. Since there are equal number of positive and negative links, a good predictor would rank a high amount of links in the top half. The neural network approaches performed vastly better in those cases, although the varying amount of such positives affected the overall effect.

For the MATADOR experiment, approximately 2% of the positive links had no prior common neighbours. Common Neighbours ranked none of these links in the top half of the rankings, but the best neural predictor ranked 26% there. In the BioGRID random-sliced experiment, approximately 16% of the positive links had no prior common neighbours. Common Neighbours ranked about 11% of these links in the top half, while the best neural predictor ranked 71% in the top half. For the time-sliced version, approximately 28% of the positive links had no prior common neighbours. Common Neighbours ranked about 21% of these links in the top half of the rankings, while the best neural predictor ranked 69% there. In the PubTator random-sliced experiment, approximately 2% of the positive links had no prior common neighbours. Common Neighbours ranked none of these links in the top half, while the best neural predictor ranked 51% there. For the time-sliced version, approximately 21% of the positive links had no prior common neighbours. Common Neighbours ranked about 11% of these links in the top half, while the best neural predictor ranked 57% there.

In general, for the neural network approaches, concatenate and average were the best node embedding combination techniques. Common Neighbours was the best baseline approach especially as graphs increased in size and remains quite an accurate heuristic for link prediction. In cases where the purpose of link prediction is to get only the very best links across the entire graph, then it almost does not matter which approach is chosen for a small enough *k*, but if the quality of links at higher recall levels or the performance of the predictor across most nodes is essential, the choice of method is an important factor and the neural network approaches are clearly superior if they have enough data.

The results showed that link prediction is a complex task which requires comprehensive experiments to determine best approaches, that performance is dependent on several things including the size of the graph and how it is split and that it is necessary to discern how a particular approach is achieving performance. It also highlighted that link prediction ought to be evaluated according to its intended purpose and that AUC metrics may not capture when and how well a particular approach works.

## Conclusions

In this work we investigated how node embeddings created with four graph embedding algorithms and combined with various methods perform on link prediction in biomedical graphs, with a neural link predictor. We tested in settings where links were randomly removed and where links are removed by time-slicing. We compared these methods to the performance of established baseline methods and reported performance on five metrics which aim to capture different facets of a link predictor’s performance.

Our findings in both random- and time-sliced experiments indicate that where there is enough data for the neural network methods to learn good representations and there is a negligible amount of disconnected nodes, those approaches could perform much better than the baselines. However if the graph is small or there are large amounts of disconnected nodes, existing baselines such as Common Neighbours are a justifiable choice for link prediction. At low recall levels the approaches are basically equal, but at higher recall levels across all nodes and average performance at individual nodes, then the neural network approaches are clearly superior if they have enough data. We found evidence that the neural network methods do especially well in links which feature nodes with no previous common neighbours. We also found that while in general neural network methods benefit from large amounts of data, they require considerable amounts of computational resources to scale to large datasets. These findings provide large-scale comparisons and analyses that informs and explains the best approaches to link prediction and highlight areas of further development.

The neural network approaches to link prediction provide a truly promising way forward but they are not the best in all conditions and introduce added experimental considerations such as the creation of negatives and the combination of node representations. It is also well-known that the success of neural network methods greatly rely on hyperparameter tuning.

For future work we wish to investigate the problem of creating good negatives for using machine learning methods for link prediction. Randomly creating negatives is experimentally valid but may create negatives which are not reflective of real-world difficulty. The problem of maintaining a large ratio of negative to positive links, as is the case in the real-world, without being computationally prohibitive is also worth exploring.

## Additional file

Additional file 1 Results from embedding combination methods explained but not shown in the main manuscript (Hadamard, Weighted-L1 and Weighted-L2). Some analysis on these results. It also contains additional results from the SDNE node embedding creation method explained in the paper. The results in this document also contain the standard deviation from the means reported in the main manuscript. There are also additional Precision at *k* values for *k* = 10, 20 and 40. (PDF 74 kb)

## Abbreviations

AA: Adamic-adar
CN: Common neighbours
DTI: Drug-target interaction
JI: Jaccard Index
LBD: Literature-based discovery
PPI: Protein-protein interaction
ReLU: Rectified linear unit
