# Supplementary material for: Neural networks for link prediction in realistic biomedical graphs: a multi-dimensional evaluation of graph embedding-based approaches
Source: BMC Bioinformatics. 2018 May 21;19:176. doi: 10.1186/s12859-018-2163-9 (PMC5963080; doi:10.1186/s12859-018-2163-9)
Supplement: Supplementary file 1 — Results from embedding combination methods explained but not shown in the main manuscript (Hadamard, Weighted-L1 and Weighted-L2). Some analysis on these results. It also contains additional results from the SDNE node embedding creation method explained in the paper. The results in this document also contain the standard deviation from the means reported in the main manuscript. There are also additional Precision at k values for k = 10, 20 and 40. (PDF 74 kb) [file 12859_2018_2163_MOESM1_ESM.pdf]

Supplementary Document for 'Neural Networks for Link Prediction in Realistic Biomedical Graphs: A Multidimensional Evaluation of Graph Embedding-based Approaches'

Gamal Crichton, Yufan Guo, Sampo Pyysalo and Anna Korhonen

1 Introduction

This document is supplementary to the paper: *Neural Networks for Link Prediction in Realistic Biomedical Graphs: A Multidimensional Evaluation of Graph Embedding-based Approaches*. It contains additional results and analysis which were left out of the main paper due to space constraints.

For SDNE, two implementations were tried: the one created by the authors (Wang et al., 2016) and one created by (Goyal and Ferrara, 2017). We used the parameters from (Goyal and Ferrara, 2017) because our attempted hyper-parameters did not give good results and, though we contacted both sets of authors, only they responded to our request for the hyper-parameters used in their experiments.

2 Results and Discussion

In the result tables, the number in **bold** represent the best score for a particular metric. The difference between the best and scores with an asterisk (*) are not statistically significant.

2.1 MATADOR

These results are in Table 1. The additional result is that SDNE is much worse than the other approaches for this dataset. This may be due to the fact that it is the deepest of all the neural network approaches and so required more data to train properly. In the main paper, we already attribute the relatively poor performance of the deep learning models compared to the baselines to the small size of this dataset - that argument would hold even more so for SDNE.

Note also that LINE embeddings combined with Hadamard were on par with the best performer for precision at *k*.

2.2 BioGRID

The randomly sliced experiments on this dataset are in Table 2 and the time-sliced experiments are in Table 3.

2.2.1 Random-Slice

Node2vec embeddings combined with Hadamard were on par with the best performer for precision at k.

2.2.2 Time Slice

The 'Link prediction setting' section of the paper explains why it is more difficult to perform link prediction in the time-slice setting. To recap: first, new nodes can be introduced to the graph at later time periods which will present little or no information to the link predictor to use as they will have no links to other nodes in the time period which the predictor uses to make predictions .Second ,in evolving graphs the easier links tend to form first and more difficult ones later, so the edges to be predicted in later time periods tend to be more difficult.

As expected, the majority of the approaches performed worse in all metrics than the randomly sliced experiments with this dataset. However there were some exceptions. DeepWalk embeddings combined by Weighted-L1 and L2, node2vec embeddings combined with Weighted-L1 and all baselines recorded

Method	Node Combi- nation	AUC (ROC)	AUC (PR)	MAP	Avg. R-prec	Prec @ *k*
Deep-Walk	Average	95.93 ± .003	95.82 ± .005	89.81 ± .003	86.86 ± .003	98.77 ± .004*
	Concat	94.97 ± .004	94.83 ± .003	88.30 ± .0003	84.63 ± .0009	98.34 ± .002*
	Hadamard	90.21 ± .003	91.55 ± .004	86.65 ± .01	82.59 ± .01	97.56 ± .005
	W-L1	80.45 ± .01	82.74 ± .01	69.27 ± .006	62.56 ± .0004	93.74 ± .02
	W-L2	85.67 ± .0009	88.12 ± .004	77.31 ± .004	71.57 ± .005	97.44 ± .004
LINE	Average	80.63 ± .01	81.30 ± .006	67.74 ± .02	61.04 ± .03	91.65 ± .009
	Concat	81.16 ± .01	81.82 ± .007	68.53 ± .02	61.42 ± .02	92.00 ± .009
	Hadamard	89.11 ± .008	90.37 ± .006	83.45 ± .01	77.47 ± .02	98.00 ± .003
	W-L1	70.76 ± .02	79.32 ± .009	73.86 ± .007	66.15 ± .006	98.02 ± .009*
	W-L2	69.52 ± .02	76.37 ± .01	70.94 ± .003	63.33 ± .0006	92.38 ± .02
node-2vec	Average	78.38 ± .02	78.75 ± .02	66.42 ± .02	59.32 ± .02	88.67 ± .01
	Concat	77.62 ± .03	77.54 ± .03	65.44 ± .02	58.40 ± .02	87.25 ± .03
	Hadamard	84.74 ± .03	85.12 ± .02	82.34 ± .02	76.88 ± .02	93.71 ± .02
	W-L1	75.38 ± .05	74.98 ± .05	69.32 ± .03	62.08 ± .04	83.94 ± .05
	W-L2	74.31 ± .05	74.57 ± .05	69.56 ± .03	62.48 ± .04	84.62 ± .05
SDNE	Average	55.77 ± .02	55.22 ± .03	54.81 ± .02	47.21 ± .02	57.56 ± .05
	Concat	54.88 ± .01	54.17 ± .01	53.37 ± .01	46.14 ± .01	56.41 ± .02
	Hadamard	53.12 ± .02	52.20 ± .02	51.81 ± .01	47.85 ± .07	52.84 ± .03
	W-L1	54.35 ± .01	53.44 ± .01	50.06 ± .06	45.56 ± .03	54.93 ± .03
	W-L2	52.60 ± .01	51.34 ± .01	50.67 ± .01	43.41 ± .01	50.44 ± .01
AA	N/A	91.97 ± .001	88.40 ± .002	87.16 ± .001	85.06 ± .003	86.87 ± .006
CN	N/A	**97.27** ± **.002**	97.04 ± .003*	**95.47** ± **.002**	**94.64** ± **.002**	98.74 ± .004*
JC	N/A	97.23 ± .002*	**97.10** ± **.001**	94.72 ± .002	92.29 ± .002	**98.96** ± **.002**

Table 1: MATADOR random-slice results

better performance for MAP. DeepWalk embeddings combined by Weighted-L1 and L2, node2vec embeddings combined with Weighted-L1 and Adamic-Adar recorded better performance for averaged R-precision. Adamic-Adar also recorded increased performance for precision at k. There are several possible contributing factors here.

For MAP and averaged R-precision, if a particular node has no positives it is removed from the calculations as these metrics are only concerned with predicted true positives. In the time-sliced data, there are a much higher percentage of nodes which have no true positives in the test slice than is the case with randomly-sliced data. These nodes are also likely to have a small amount of links and are thus difficult nodes to perform well on, so it is not surprising that the approaches which performed poorest on the randomly-sliced version of this dataset benefited from having less and easier nodes in the evaluation. The poor embeddings created for this setting as explained above would contribute to decreased performance for the other methods but as all combination methods use the same embeddings, there is something about the DeepWalk embeddings combined with Weighted L1 and L2 which help in this setting.

Node2vec embeddings combined with Hadamard had performance that was not significantly worse than the best for AUPRC and precision at k.

2.3 PubTator

The randomly sliced experiments on this dataset can be seen in Table 4 and the time-sliced experiments can be seen in Table 5.

2.3.1 Random-Slice

Nothing much to add here except to note that Common Neighbours outperformed the lower neural network performers (Hadamard, Weighted-L1 and Weighted-L2) for most metrics.

Method	Node Combi- nation	AUC (ROC)	AUC (PR)	MAP	Avg. R-prec	Prec @ *k*
DeepWalk	Average	97.69 ± .0003	97.62 ± .0006	79.24 ± .003	73.86 ± .003	99.30 ± .0007
	Concat	97.74 ± .001	97.65 ± .002	82.48 ± .006	77.70 ± .006	99.18 ± .002
	Hadamard	95.76 ± .001	96.54 ± .0005	79.63 ± .0005	74.87 ± .0007	99.25 ± .0007
	W-L1	79.17 ± .004	80.57 ± .004	51.96 ± .008	46.50 ± .009	91.71 ± .005
	W-L2	79.73 ± .002	81.08 ± .001	52.81 ± .002	47.39 ± .003	92.12 ± .001
LINE	Average	98.10 ± .002*	97.80 ± .002*	83.13 ± .02*	78.22 ± .02*	99.54 ± .002*
	Concat	98.08 ± .0003	97.76 ± .0003	82.94 ± .004	78.04 ± .009	99.29 ± .0009
	Hadamard	94.45 ± .002	95.35 ± .002	80.17 ± .0008	75.17 ± .01	99.30 ± .002
	W-L1	92.41 ± .006	92.06 ± .006	70.88 ± .009	65.21 ± .008	97.07 ± .003
	W-L2	91.80 ± .006	91.55 ± .006	71.80 ± .003	66.39 ± .005	96.56 ± .005
node2vec	Average	98.32 ± .002*	97.97 ± .03*	85.70 ± .006*	81.17 ± .005*	99.38 ± .002*
	Concat	**98.51 ± .0007**	**98.26 ± .03**	**86.49 ± .009**	**81.84 ± .009**	99.49 ± .0009*
	Hadamard	97.19 ± .001	97.17 ± .03	81.53 ± .01	76.54 ± .01	99.33 ± .002*
	W-L1	92.02 ± .007	92.30 ± .03	64.24 ± .01	59.45 ± .008	97.45 ± .003
	W-L2	93.07 ± .003	93.01 ± .03	67.11 ± .007	61.94 ± .005	97.47 ± .005
AA	N/A	86.10 ± .0003	90.75 ± .0005	70.97 ± .0008	57.65 ± .0006	96.13 ± .001
CN	N/A	91.20 ± .0004	94.96 ± .0001	75.72 ± .0004	69.81 ± .003	**99.64 ± .0002**
JI	N/A	90.80 ± .0004	93.95 ± .0003	73.93 ± .001	68.79 ± .001	98.59 ± .0002

Table 2: BioGRID random-slice results

2.3.2 Time Slice

As with the BioGRID data, the majority of the approaches performed worse in this setting than the random-sliced one, and there were again some exceptions. DeepWalk embeddings combined by Weighted-L1 and L2 had better performance in all metrics and Adamic-Adar again recorded increased performance for precision at k. Similar explanations hold for this situation as well. In this case only the DeepWalk vectors were better and they were better in all metrics and the previous explanations pertained only to the node-level metrics. These results provide strong indication that DeepWalk embeddings combined with Weighted-L1 and Weighted-L2 perform better in the time sliced setting than the random slice one, but their performances are still significantly worse than the best performers in these settings.

3 Additional K values for Precision at k

The main manuscript lists results for precision at k when k=30% of all positives. Here we add additional results fro k= 10, 20 and 30.

References

Palash Goyal and Emilio Ferrara. 2017. Graph embedding techniques, applications, and performance: A survey. *arXiv preprint arXiv:1705.02801*.

Daixin Wang, Peng Cui, and Wenwu Zhu. 2016. Structural deep network embedding. In *Proceedings of the 22nd ACM SIGKDD International Conference on Knowledge Discovery and Data Mining*, KDD '16, pages 1225–1234, New York, NY, USA. ACM.

Method	Node Combi-nation	AUC (ROC)	AUC (PR)	MAP	Avg. R-prec	Prec @ *k*
Deep-Walk	Average	89.40 ± .009	90.10 ± .01	68.94 ± .001	63.30 ± .001	97.25 ± .001*
	Concat	92.12 ± .004	92.78 ± .003	71.61 ± .002	65.96 ± .002	98.04 ± .002
	Hadamard	89.03 ± .004	91.39 ± .004	66.28 ± .002	60.34 ± .003	98.31 ± .003
	W-L1	69.75 ± .02	67.43 ± .01	59.74 ± .006	54.61 ± .006	73.26 ± .006
	W-L2	72.11 ± .01	69.33 ± .006	59.84 ± .004	54.51 ± .005	75.02 ± .005
LINE	Average	91.86 ± .006	92.31 ± .006	72.85 ± .002	67.76 ± .002	97.40 ± .002
	Concat	93.55 ± .003	93.74 ± .002	73.60 ± .002	68.57 ± .002	97.90 ± .002
	Hadamard	77.70 ± .02	82.51 ± .01	67.78 ± .004	61.33 ± .005	96.05 ± .005
	W-L1	82.36 ± .007	81.32 ± .009	66.66 ± .004	60.93 ± .005	88.54 ± .005
	W-L2	79.79 ± .03	78.82 ± .02	66.53 ± .002	60.75 ± .004	86.76 ± .004
node-2vec	Average	**95.25 ± .002**	**95.43 ± .004**	74.91 ± .001	**70.39 ± .0006**	98.26 ± .0006
	Concat	93.66 ± .002	94.66 ± .004*	73.48 ± .002	68.77 ± .002	98.40 ± .002*
	Hadamard	93.94 ± .002	94.02 ± .009*	71.81 ± .003	66.57 ± .003	97.59 ± .003*
	W-L1	89.06 ± .002	88.70 ± .004	66.17 ± .005	61.20 ± .004	93.86 ± .004
	W-L2	88.81 ± .003	88.43 ± .006	66.09 ± .01	61.02 ± .01	93.54 ± .01
AA	N/A	77.46 ± .00006	87.69 ± .0003	74.84 ± .0003	61.39 ± .001	98.10 ± .0004
CN	N/A	85.07 ± .0001	91.81 ± .0003	**76.20 ± .001**	67.73 ± .004	**99.38 ± .0002**
JC	N/A	84.74 ± .0002	90.20 ± .0006	75.60 ± .001	67.49 ± .0003	97.45 ± .0007

Table 3: BioGRID time-slice results

Method	Node Combi-nation	AUC (ROC)	AUC (PR)	MAP	Avg. R-prec	Prec @ *k*
Deep-Walk	Average	98.85 ± .03	99.01 ± .02	83.67 ± .12	75.97 ± .28	99.93* ± .006
	Concat	**99.20 ± .006**	**99.30 ± .006**	**91.01 ± .16**	85.46 ± .20	99.94* ± .006
	Hadamard	98.44 ± .06	98.68 ± .03	84.67 ± .36	77.84 ± .31	99.88 ± .01
	W-L1	88.96 ± .40	89.63 ± .36	60.76 ± 1.7	51.21 ± 1.5	97.64 ± .16
	W-L2	89.25 ± .01	89.90 ± .07	62.10 ± .36	52.57 ± .40	97.67 ± .16
LINE	Average	99.10 ± .09*	99.23 ± .08*	90.36 ± .82*	84.56 ± 1.0	**99.97 ± .03**
	Concat	99.13 ± .02	99.24 ± .02	90.07 ± .34	84.03 ± .48	99.95 ± .006*
	Hadamard	98.30 ± .04	98.49 ± .05	86.40 ± .69	79.28 ± .87	99.90 ± .006
	W-L1	93.93 ± .10	94.16 ± .10	78.25 ± .94	69.48 ± 1.1	98.97 ± .13
	W-L2	94.23 ± .11	94.51 ± .02	77.97 ± .96	69.00 ± 1.2	99.13 ± .06
node-2vec	Average	98.71 ± .05	98.90 ± .04	82.98 ± .58	75.29 ± .72	99.94 ± .006*
	Concat	99.16 ± .03*	99.21 ± .02	88.94 ± .29	82.14 ± .30	99.92 ± .0*
	Hadamard	98.81 ± .03	98.91 ± .02	86.40 ± .22	79.07 ± .27	99.87 ± .006
	W-L1	88.07 ± .03	87.28 ± .11	87.28 ± 1.4	48.95 ± 1.4	94.08 ± .16
	W-L2	88.85 ± .07	88.26 ± .02	88.26 ± .74	50.72 ± .69	94.90 ± .13
AA	N/A	92.92 ± .03	84.56 ± .04	56.48 ± .16	66.38 ± .13	83.33 ± .02
CN	N/A	98.40 ± .01	98.28 ± .01	79.84 ± .19	**87.10 ± .16**	99.94 ± .00*
JI	N/A	92.36 ± .02	87.59 ± .03	65.44 ± .05	59.74 ± .04	91.21 ± .01

Table 4: PubTator random-slice results

Method	Node Combi- nation	AUC (ROC)	AUC (PR)	MAP	Avg. R-prec	Prec @ *k*
Deep-	Average	93.86 ± .002*	95.51 ± .002*	70.78 ± .004*	62.16 ± .004*	**99.89 ± .0001**
Walk	Concat	**93.99 ± .002**	**95.70 ± .001**	**71.11 ± .003**	**62.65 ± .003**	**99.89 ± .00**
	Hadamard	87.23 ± .002	91.33 ± .001	54.72 ± .002	46.22 ± .002	99.70 ± .0005
	W-L1	92.06 ± .0005	93.23 ± .0002	66.47 ± .001	57.29 ± .0008	98.77 ± .0003
	W-L2	91.81 ± .002	93.06 ± .002	65.89 ± .003	56.66 ± .004	98.76 ± .0003
LINE	Average	88.68 ± .03*	92.27 ± .02*	55.61 ± .09*	46.41 ± .09*	**99.89 ± .0002**
	Concat	90.32 ± .005	93.01 ± .002	62.51 ± .02	53.21 ± .02	**99.89 ± .0006**
	Hadamard	87.09 ± .007	89.98 ± .005	51.97 ± .01	42.43 ± .01	99.10 ± .003
	W-L1	83.58 ± .0005	86.55 ± .004	47.71 ± .003	38.11 ± .002	97.26 ± .007
	W-L2	82.81 ± .003	85.79 ± .003	47.07 ± .005	37.49 ± .004	96.78 ± .006
node-	Average	88.40 ± .003	92.07 ± .002	55.72 ± .003	46.48 ± .004	99.87 ± .00006
2vec	Concat	88.13 ± .0006	91.83 ± .0002	53.24 ± .002	43.69 ± .004	99.84 ± .0001
	Hadamard	85.24 ± .001	90.63 ± .001	47.76 ± .003	38.84 ± .003	99.81 ± .0005*
	W-L1	84.68 ± .003	89.08 ± .001	44.69 ± .003	35.34 ± .003	98.57 ± .00
	W-L2	84.48 ± .0008	89.12 ± .0004	44.68 ± .0005	35.49 ± .0002	98.67 ± .0004
AA	N/A	85.10 ± .0002	80.24 ± .0003	35.49 ± .0003	40.13 ± .0002	90.56 ± .0005
CN	N/A	88.37 ± .00006	88.83 ± .00006	43.67 ± .0001	46.59 ± .0002	99.84 ± .00008
JI	N/A	86.08 ± .0002	83.52 ± .0004	38.66 ± .0002	38.75 ± .0009	94.27 ± .00004

Table 5: PubTator time-slice results

Method	Node Combination	P@10	P@20	P@40
Deep-	Average	99.47	99.04	98.26
Walk	Concat	99.65	98.87	98.22
	Hadamard	98.61	98.26	98.22
	W-L1	98.61	98.87	91.66
	W-L2	98.78	98.87	96.61
LINE	Average	93.03	91.98	88.51
	Concat	93.73	93.12	89.60
	Hadamard	92.33	90.33	86.55
	W-L1	98.26	98.34	98.12
	W-L2	95.12	93.12	89.60
node-	Average	89.91	89.40	84.75
2vec	Concat	92.35	89.57	86.62
	Hadamard	95.65	94.01	90.36
	W-L1	92.17	91.49	86.53
	W-L2	94.43	92.79	87.92
SDNE	Average	57.04	54.96	54.00
	Concat	55.83	53.30	52.00
	Hadamard	55.83	55.91	53.91
	W-L1	53.22	53.57	53.26
	W-L2	50.96	48.61	49.61
AA	N/A	61.32	66.18	73.88
CN	N/A	97.49	98.36	97.10
JC	N/A	97.10	98.07	97.54

Table 6: MATADOR additional P@K results

Method	Node Combination	P@10	P@20	P@40
Deep-Walk	Average	99.61	99.50	99.23
	Concat	99.69	99.59	99.33
	Hadamard	99.42	99.39	99.25
	W-L1	97.68	94.00	87.12
	W-L2	97.29	94.74	89.30
LINE	Average	99.48	99.37	99.14
	Concat	99.63	99.57	99.27
	Hadamard	99.56	99.37	98.94
	W-L1	99.11	98.36	96.81
	W-L2	98.90	97.59	95.90
node-2vec	Average	99.61	99.54	99.25
	Concat	99.62	99.53	99.29
	Hadamard	99.31	99.28	99.02
	W-L1	98.24	97.97	97.35
	W-L2	98.11	97.70	96.90
AA	N/A	93.52	94.83	96.47
CN	N/A	99.79	99.72	99.56
JC	N/A	98.21	98.49	98.45

Table 7: BioGRID additional P@K results

Method	Node Combination	P@10	P@20	P@40
Deep-Walk	Average	99.12	98.67	97.36
	Hadamard	97.99	97.22	95.36
	W-L1	98.48	97.79	96.39
	W-L2	98.55	97.94	96.59
LINE	Average	99.08	98.59	97.16
	Hadamard	95.62	94.45	92.06
	W-L1	96.84	94.89	90.48
	W-L2	96.87	95.32	91.14
AA	N/A	85.62	88.39	92.17
CN	N/A	99.10	98.60	96.92
JC	N/A	82.32	84.89	86.67

Table 8: PubTator additional P@K results
